# Multimodular Assessment of a Traumatic Bone Cyst Overlapped with Apical Periodontitis

**DOI:** 10.1155/2020/8829305

**Published:** 2020-11-25

**Authors:** Davide Musu, Giulia Bardini, Hagay Shemesh, Claudia Dettori, Elisabetta Cotti

**Affiliations:** ^1^Department of Conservative Dentistry and Endodontics, University of Cagliari, Italy; ^2^Department of Endodontology, Academic Centre for Dentistry Amsterdam (ACTA), Amsterdam, Netherlands

## Abstract

Traumatic bone cyst (TBC), a “pseudocyst” that usually affects long bones, is a rare lesion among cystic lesions in the jaws. The most commonly affected site is the posterior mandible. Most of the time, TBC is asymptomatic and discovered during routine radiographic examination. The treatment recommended for TBC is surgical exploration followed by curettage of the bony walls, which also serves as a diagnostic procedure. A 27-year-old Caucasian male with a noncontributory medical history was referred to our department for the endodontic evaluation of the mandibular right first and second molars, which were connected to an extensive asymptomatic osteolytic lesion. A multimodular diagnostic assessment involving CBCT imaging, ultrasound, and histopathologic examination led to a definite diagnosis of a TBC overlapping with apical periodontitis (AP). Subsequently, a multidisciplinary treatment approach was performed, including surgical excision and biopsy of the lesion, endodontic retreatment of the right mandibular first molar, and postsurgical root canal treatment of the second molar. During the follow-up period of five years, the patient was reassessed periodically once a year and showed, in the absence of signs and symptoms, progressive healing of the affected area. The present article reports a case following the CARE guidelines of a TBC combined with AP where a multimodular diagnostic assessment was performed and discusses the possible pathogenetic mechanisms involved in its generation.

## 1. Introduction

Although the majority of osteolytic lesions in the periradicular area of teeth are inflammatory in origin, some may not be inflammatory. The assessment of osteolytic lesions in the maxillary bones should always involve an exhaustive medical history report and a careful clinical examination comprising diagnostic tests and radiographic examinations that can be crucial in the differential diagnosis between apical periodontitis (AP) and nonendodontic lesions [1]. Three-dimensional imaging systems provide additional information on the extension of the lesions in the maxillary bones, their relationships with the surrounding anatomical structures and the aggressiveness of the disease, [2] while ultrasound examination with color-power Doppler can detect the content of the pathologic cavity (i.e., solid and empty/fluid filled) and its vascular supply [3]. Traumatic bone cysts (TBCs) are rare lesions that constitute 0.2 to 0.9% of all cystic lesions in the jaws, where the site most commonly affected is the posterior mandible. It is often diagnosed during the first two decades of life with an even distribution among the sexes [4]. TBC usually affects long bones and is defined as “an intraosseous cyst having a tenuous lining of connective tissue with no epithelium” and consequently a “pseudocyst” [5]. Most of the time, TBC is asymptomatic and discovered during routine radiographic examination; alternative pain is the most common symptom, together with tooth sensitivity, paresthesia, and painless swelling. Despite its name, a clear history of trauma in TBC is often questionable [5, 6]. The treatment recommended for TBC is surgical exploration followed by curettage of the bony walls, which also serves as a diagnostic procedure [4–7]. The purpose of the present article is to report a case following the CARE guidelines [8] of a TBC combined with AP, where a multimodular diagnostic assessment was performed and to discuss the possible pathogenetic mechanisms involved in its generation.

## 2. Case Presentation

**Figure 1:**
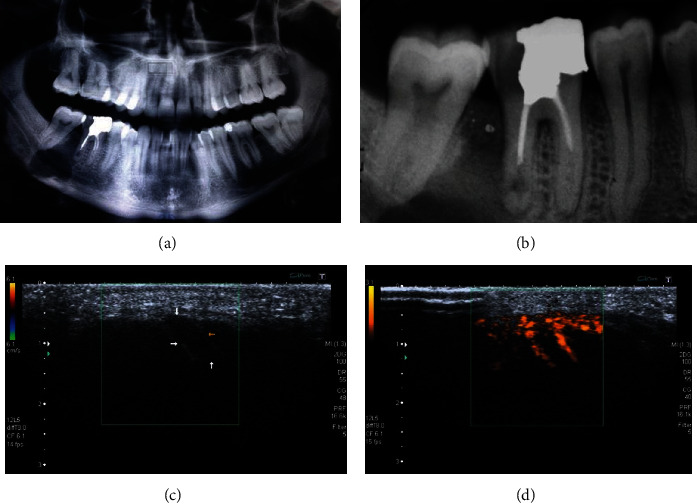
Images of the 27-year-old patient presenting an extensive asymptomatic lesion of the right posterior mandible. (a) The panoramic radiograph shows a well-defined unilocular osteolytic lesion with sclerotic margins located between the two molar teeth and superimposed on the alveolar inferior nerve. (b) The periapical radiograph of the first molar depicting inadequate root canal treatment with signs of perforation in the apical third of the distal root involved in the osteolytic lesion. (c) B-mode ultrasound examination showing a transonic lesion (arrows). (d) Power-Doppler image showing the presence of peripheral vascularity.

**Figure 2:**
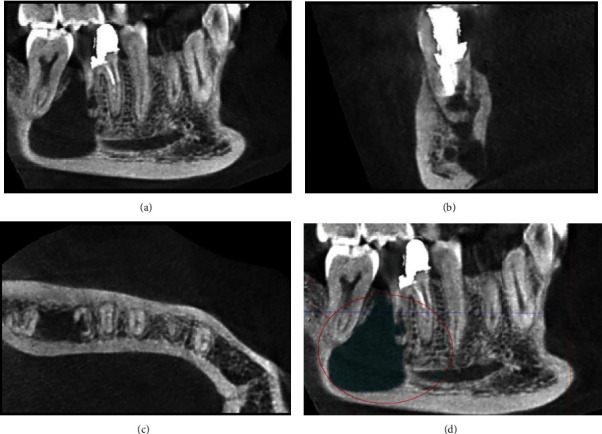
(a) CBCT revealing a well-defined unilocular lesion between the second and first right mandibular molars. (b) Sagittal view showing the expansion of the lesion toward the lingual wall and the consequent thinning of the lingual plate. (c) Axial section showing a perforation in the distal and lingual aspects of the apical third of the distal root of the right first molar. (d) The main osteolytic lesion appears in continuity with the perforation in the distal root of the right mandibular first molar.

**Figure 3:**
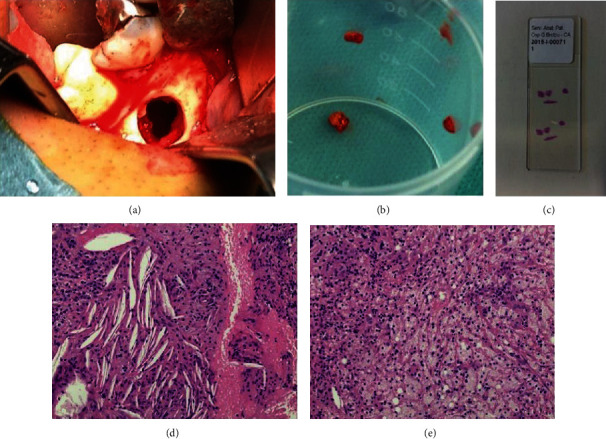
(a) Intraoperative view showing the bone cavity filled with serum sanguineous fluid. (b) Gross specimens of small dimensions collected for histopathological examination. (c) Hematoxylin-eosin histology slide containing the tissue fragments. (d) Photomicrograph shows the lesion to be composed of fibrosclerotic tissue with calcifications and cholesterol clefts and the absence of epithelium. (e) Histopathological section revealing the presence of a chronic inflammatory infiltrate with numerous foamy histiocytes and multinucleated giant cells.

**Figure 4:**
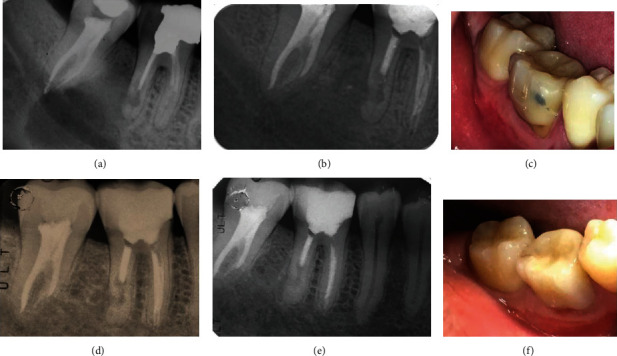
(a) Postoperative periapical radiograph showing root canal treatment of the second mandibular molar. (b) Four-month periapical radiograph showing an increase in radiopacity and trabeculae formation in the area of the intervention. (c) Four-month clinical photograph. (d) Two-year follow-up periapical radiograph. (e) Five-year follow-up periapical radiograph showing both repair of the distal root and bone healing. (f) Clinical photograph at the five-year recall visit.

**Figure 5:**
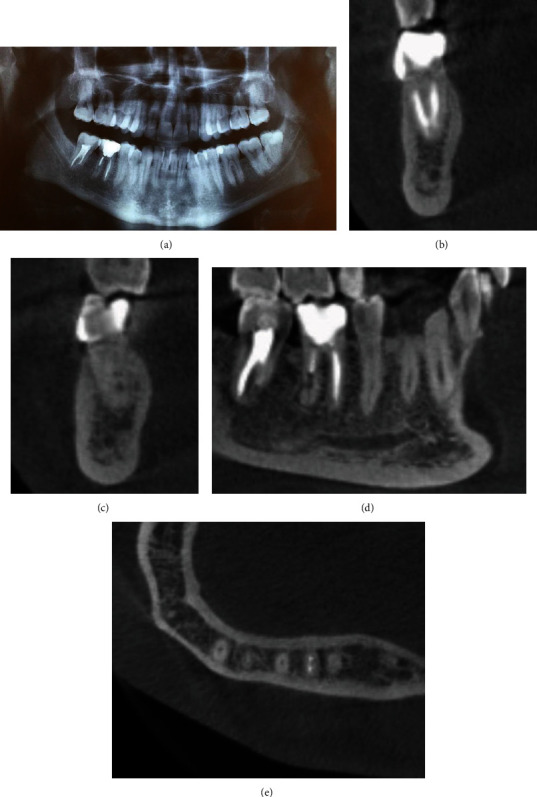
Panoramic radiograph showing complete healing and absence of recurrence after five years. (b–d) Five-year CBCT examination showing complete healing of the lesion. (c–e) The sagittal and axial views demonstrate remineralization of the area around and within the perforated root and depict the complete formation of the vestibular and lingual cortical plates.

A 27-year-old Caucasian male with a noncontributory medical history and no previous trauma was referred from the maxillofacial department of the hospital for the endodontic evaluation of the mandibular right first and second molars, which were connected to an extensive asymptomatic osteolytic lesion incidentally discovered on a routine panoramic radiograph and scheduled to be treated surgically under general anesthesia. The patient reported that he had experienced pain and swelling in the right mandible months before, while the first molar had a history of caries, extensive amalgam restoration, and incongruous endodontic treatment performed ten years earlier. The clinical examination showed no sign of buccal or lingual bone expansion, no lymph node involvement, and intact overlaying mucosa. The first mandibular molar was asymptomatic. The right second mandibular molar was asymptomatic with an intact crown, and it responded normally to the sensitivity tests at the time of our examination. The panoramic radiograph showed a well-defined unilocular osteolytic lesion with sclerotic margins located between the two molar teeth and superimposed on the alveolar inferior nerve with a slight displacement of the second molar. The first molar showed inadequate root canal treatment with signs of resorption in the apical third of the distal root involved in the osteolytic lesion. A preoperative periapical radiograph taken using the paralleling technique confirmed these findings (Figure 1). Cone beam computed tomography (CBCT) revealed a well-defined unilocular lesion, with considerable expansion toward the lingual wall and consequent thinning of the lingual plate. From the axial and sagittal sections, it was possible to diagnose a perforation both in the distal and lingual aspects of the apical third of the distal root of the right first molar (Figure 2). To assess the content and vascularity of the lesion, a real-time ultrasound examination with the application of color-power-Doppler (CPD) was performed using a Toshiba Aplio XG (Toshiba Medical Systems, Crawley, UK) apparatus with a regular size, linear, high definition, and multifrequency ultrasound probe at 8-12 MHz. The exam displayed a transonic, fluid-filled lesion with a well-defined hyperechoic bone contour, perilesional vascularity, and no internal vascular supply, suggestive of a cystic lesion (Figure 1). The diagnosis, based on all the exams performed, was a nonendodontic cystic lesion of the right mandible in the area of the first and second mandibular molars and AP in a previously treated first mandibular molar with a perforation in the distal root. The treatment plan was discussed with the patient and comprised the following steps: (1) surgical excision and biopsy of the lesion for the histopathologic evaluation; (2) endodontic retreatment of the right mandibular first molar, with the repair of the perforating defect on the distal root; (3) a possible postsurgical root canal treatment of the second molar. Surgery was performed in the maxillofacial clinic under general anesthesia after an inferior alveolar nerve block together with infiltration with anesthetic of the surrounding tissues. A mucoperiosteal flap was raised exposing the buccal bone, which did not present any sign of resorption or expansion. A bone window was created using a surgical bur with continuous water cooling to reach the lesion (Figure 3); the disclosed bone cavity had the size and shape shown by CBCT and was filled with serum/blood fluid, as seen in the ultrasound exam; thus, a temporary intraoperative diagnosis of TBC was made. The fluid was evacuated, and the walls of the lesion were carefully explored and curetted; however, only a few specimens of tissue were collectable for histopathologic examination. After the formation of the blood clot, the flap was repositioned and sutured. Histologic examination of the tissue fragments reported fibrosclerotic tissue with calcifications and cholesterol clefts, extensively interested in a chronic inflammatory infiltrate with numerous foamy histiocytes and foreign body–like multinucleated giant cells (Figure 3). Four weeks later, the patient was asymptomatic; however, at the postsurgical endodontic assessment, the second molar did not respond to the sensitivity tests, which was due to a possible resection of the alveolar neurovascular bundle, and a diagnosis of pulp necrosis was made. After informed consent was obtained, local anesthesia was administered using mepivacaine 2% 1 : 100 000 epinephrine followed by isolation of the teeth using a rubber dam. Root canal treatment of the second molar was performed in 1 appointment without complications. Two weeks later, the patient was still asymptomatic, and secondary treatment was performed on the first molar. After informed consent, local anesthesia administration, rubber dam isolation, and root canal retreatment of the mesial canals were achieved without obstacles, while the working length determined with the apex locator in the distal root canal was considerably shorter than the length of the root because of perforation, which was established by measuring the CBCT scans. The canals were instrumented manually and irrigated with 5.25% NaOCl and a final rinse of 17% EDTA solution. The apical half of the distal canal was dried with paper points and obturated using Ortho-MTA (BioMTA, Seoul, Republic of Korea) applied with a carrier to seal the perforating defect, while the remaining coronal half was filled with flowable gutta-percha and the tooth restored with composite (Figure 4). Four months later, the patient was asymptomatic, and radiographic control showed an increase in radiopacity and trabeculae formation in the bone (Figure 4). The patient attended the periodical recall visits, and at the five-year follow-up was asymptomatic. The radiographic examination showed complete healing of the lesion, with remineralization of the area around and within the perforated root. An additional CBCT examination depicted the complete formation of the vestibular and lingual cortical plates (Figures 4 and 5).

## 3. Discussion

The present report documents a case of a mandibular lesion diagnosed as TBC accompanied by complications, such as involvement in the field of a tooth affected by AP and an inflammatory granuloma-like histological pattern. TBC is described with a variety of names (simple bone cyst, hemorrhagic bone cyst, solitary bone cyst, extravasation cyst, and unicameral bone cyst), suggesting a lack of complete understanding of its nature [9]. Indeed, several possible theories have been formulated for its etiology and pathogenesis [6, 10–12], as reported in Table 1. The radiographic features of a TBC are those of a unilocular, well-circumscribed, radiolucent lesion with or without sclerotic margins, which may extend between the roots of teeth that are normally vital with the lamina dura preserved [7, 9]. Our CBCT findings were coherent with these descriptions, except for the loss of the lamina dura around the distal root of the first molar, a finding that validated the diagnosis of AP on that tooth. Through ultrasound real-time examination with CPD, the lesion was visualized as cystic (transonic/anechoic well-defined cavity with no evidence of central vascularization and a strong peripheral power Doppler signal to indicate a layer of vascular lining in the walls of the lesion) [13], findings that were confirmed following surgical exploration and biopsy. To our knowledge, this is the first time a TBC was assessed by ultrasound examination. According to the literature, during surgical exploration, the TBC appears as a cavity that can be either empty or filled with fluid (serum or blood), and a small amount of fibrous connective tissue can be collected occasionally while curetting the walls [9, 12, 14, 15]. Histopathologic examination usually reveals the absence of epithelial lining, presence of connective tissue, areas of vascularity with occasional chronic inflammatory cells, and scattered areas of new bone formation [14, 15]. In the present case, the small fragments collected showed highly vascularized connective tissue, confirming the echographic data. Nevertheless, the pathological analysis highlighted an inflammatory transformation of the connective wall of the lesion indistinguishable from that observed for an apical granuloma. While the cause and progression of TBC remain unclear (Table 1), trauma is still the most important etiological factor and must be considered in the greater varieties of clinical situations [6, 10–12, 15]. According to this case, different hypotheses should be considered in the pathogenesis of this lesion. First, the TBC and AP developed independently; the first was due to one of the theories presented [1–6] and the latter was due to infection of the root canal system. The contemporary expansion of the two lesions in the mandible and the resorption of the surrounding bone created communication between lesions, which may have maintained their specificities. The second hypothesis is supported by the first theory reported in Table 1. AP developed initially in the first molar and during root canal treatment of the tooth, iatrogenic perforation (trauma) generated intraosseous bleeding (theory 1). According to a third hypothesis, the low-grade, chronic infection sustained by the right mandibular first molar may have influenced the bone marrow in the vicinity, which is involved in the later pathogenesis of TBC (theory 2). A limitation of the present report relies on the decision to treat the second molar, which was due to a postsurgical diagnosis of pulp necrosis following cold sensitivity tests. Although the testing was performed four weeks after surgery, it is possible for the pulpal neural bundle to await, to require more time to recover. Fortunately, upon access to the pulp chamber, the tissues appeared ischemic, and the clinical diagnosis was confirmed. The strength of the present report was the multimodular assessment of the case where the radiographs disclosed the presence of the lesions and the previous dental treatments. The content and the features of the TBC were evaluated with echography, while CBCT was of paramount importance to determine the volume and spatial relationship of the lesion with and within the anatomical landmarks to plan the endodontic and surgical interventions and to achieve a predictable follow-up. Furthermore, the composite treatment plan involving the surgical access of the TBC and the endodontic retreatment of the right mandibular first molar affected by AP resulted in very satisfactory healing.

## 4. Conclusions

The clinical significance of this case report is the presence of two lesions, AP and TBC, combined in the same anatomical site, and such presentation has not been described before in the literature. The management of this pathological entity possibly was due to a multimodular assessment. In addition, this is the first report to describe the ultrasound examination of a combined lesion.

## Figures and Tables

**Table 1:** Etiopathogenetic theories of the TBC.

*N*°	Theory	Statement
1	Traumatic-hemorrhagic	A trauma can lead to intramedullary hemorrhage. Compromised vascular supply, edema, and aseptic bone necrosis caused by the trauma lead the blood clot to liquefy. In the area, lytic enzymes are released, and osteoclastic bone resorption is activated. The expansion of the cavity seems to be sustained by the edema and blood extravasation.
2	Infective	A small, low-grade, chronic infection of bone marrow is involved in the pathogenesis
3	Lesion degeneration	A developing tumor or lesion (i.e., hemangioma, lymphoma, fibrous-osseous dysplasia, or central giant cell granuloma) undergoes liquid degeneration, leaving behind an empty cavity.
4	Local thrombosis	A local thrombosis can generate either a local ischemia with necrosis of bone marrow and blockage of interstitial fluid drainage leading to the formation and expansion of an intraosseous cavity.
5	Developmental	A failure of mesenchymal tissue to form bone and cartilage occurs and instead generates immature synovial cavities, which coalesce to form a larger connective tissue-lined defect.
6	Systemic disease	An imbalance between osteoclastic and osteoblastic activity, parathyroid disease, or a peculiarity in vessel walls or blood coagulation can predispose to the development of a TBC.

## Data Availability

The data that support the findings of this study are available from the corresponding author upon reasonable request.
